# Total Scalp Excision and Reconstruction Using a Free Omental Flap

**Published:** 2015-11-03

**Authors:** Kyra Sierakowski, Nicholas S. Solanki, Peter Riddell

**Affiliations:** Department of Plastic & Reconstructive Surgery, Flinders Medical Centre, Bedford Park, Adelaide, South Australia, Australia

**Keywords:** omental flap, scalp reconstruction, free flap, halo, microsurgery

## DESCRIPTION

An 84-year-old man with extensive sun-damaged skin on his scalp underwent a subtotal scalp excision for squamous cell carcinoma after 5 prior incomplete resections. The large scalp defect was reconstructed with a free omental flap and skin grafts. Mayfield pins and halo were used for positioning postoperatively.

## QUESTIONS

1. **Where can omental free flaps be utilized?**
2. **What are the advantages and disadvantages of the omental free flap?**
3. **How do you manage postoperative positioning and flap monitoring?**
4. **Why was the omental free flap the reconstructive option of choice for this case?**

## DISCUSSION

The omental free flap was first described by McLean and Buncke^1^ for a large scalp defect in 1972. Since this time, it has been used for a variety of anatomical defects of the chest, abdominal wall,^2^ extremities including hand,^3^ breast,^4^ and its original application in head and neck reconstruction.^5^

The omental flap offers a large amount of malleable tissue,^6^ which is well vascularized and has the benefit of immunological properties.^7^ It also has a long and reliable vascular pedicle,^3^ which can be safely anastomosed with the superficial temporal vessels.^8^ Unlike the latissimus dorsi free flap, the omental flap avoids repositioning of the patient mid-operation. Disadvantages of the omental flap are that accessing the donor site requires opening the abdominal cavity. Historically, this meant a laparotomy by necessity; but these days, laparoscopic omental harvesting is possible. Another potential limitation is that the omentum must be in good condition and therefore prior abdominal surgery may exclude the omental flap as a reconstructive option.^2^

To avoid trauma to the newly grafted free flap, Mayfield pins were inserted at the time of surgery. This allowed a halo to be fitted to the skull, alleviating any pressure and potential necrosis of the skin grafts and the free flap. This technique was used in a similar case at the same institution 3 decades earlier, with good results.^1^ Postoperative observations of the viability of the flap are made challenging by the overlying grafts and the lack of a skin paddle. Ideally, an implantable Doppler device would be used to monitor the vascular status of the flap; however, this was unavailable. In this case, a hypodermic needle was used to prick the omental fat through a hole in the skin graft fenestration. This proved to be an adequate technique to as assess flap vascularity.

This patient had previously undergone multiple incomplete surgical resections of his scalp squamous cell carcinoma. Therefore, it was of upmost importance for this patient to achieve oncological clearance and reconstruction with a single procedure; cosmetic outcome was of secondary priority. The omental flap was thought to be the most reliable option for reconstructing the large defect with the least risk of requiring subsequent surgery. Because of the highly vascular tissue of the omentum, the split-thickness skin grafts also had a good likelihood of success.

This case reminds us of a classic reconstructive free flap; the omental free flap is a valuable tool for reconstruction of large soft-tissue defects. It is a reliable and adaptable option that should not be overlooked.

## Figures and Tables

**Figure 1:**
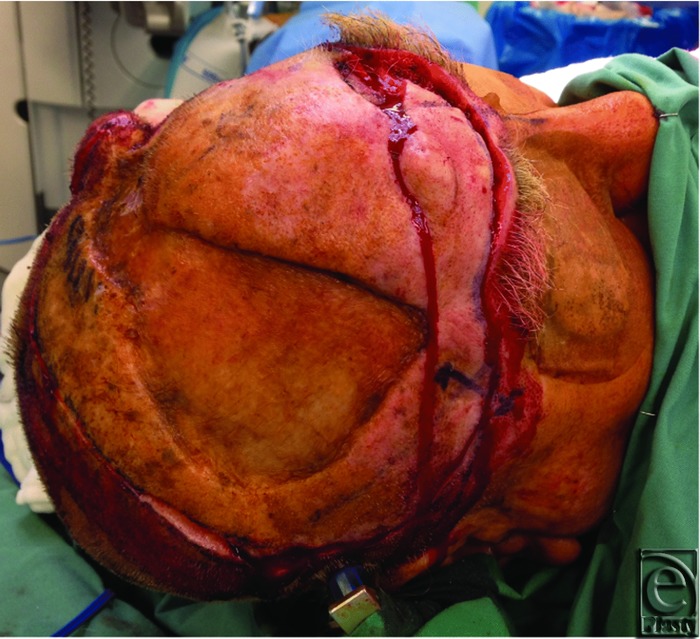
Intraoperative image demonstrating the extent of the scalp excision from the supraorbital ridge to the occiput.

**Figure 2:**
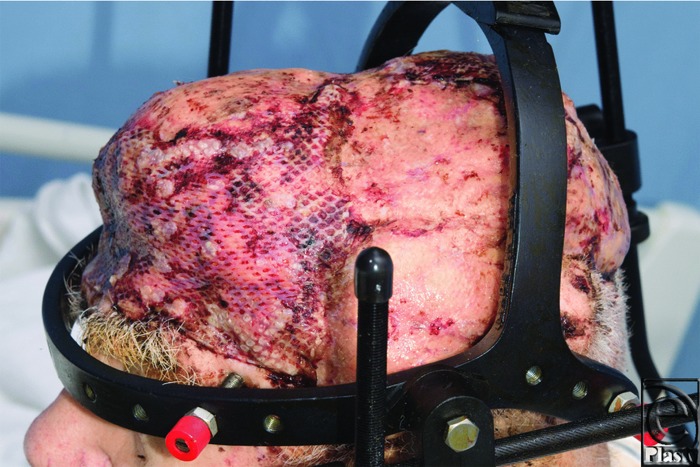
Halo in position on day 27 after the original surgery, 3 days after regrafting for areas of graft loss.

**Figure 3:**
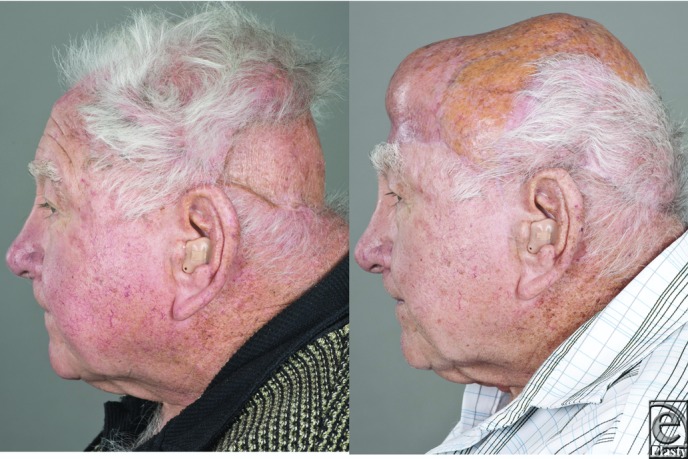
Preoperative (left) and postoperative (right) images.
